# Mediating Role of Nutrition in Psychological, Social and Physical Frailty among Hospitalized Older Adults in China

**DOI:** 10.1093/geroni/igaf122.2869

**Published:** 2025-12-31

**Authors:** Jing Ge, Ping He, Bei Cheng, Wenhan Li, Aihong Liu, Tangmeng Guo, Kemeng Zhang, Yi Zhang

**Affiliations:** Department of Geriatrics, Union Hospital, Tongji Medical College, Huazhong University of Science and Technology, 1277 Jiefang Avenue, Wuhan 430022, Hubei, China, Wuhan, Hubei, China (People’s Republic); Department of Geriatrics, Union Hospital, Tongji Medical College, Huazhong University of Science and Technology, 1277 Jiefang Avenue, Wuhan 430022, Hubei, China, Wuhan, Hubei, China (People’s Republic); Department of Geriatrics, Union Hospital, Tongji Medical College, Huazhong University of Science and Technology, 1277 Jiefang Avenue, Wuhan 430022, Hubei, China, Wuhan, Hubei, China (People’s Republic); Department of Geriatrics, Union Hospital, Tongji Medical College, Huazhong University of Science and Technology, 1277 Jiefang Avenue, Wuhan 430022, Hubei, China, Wuhan, Hubei, China (People’s Republic); Department of Geriatrics, Union Hospital, Tongji Medical College, Huazhong University of Science and Technology, 1277 Jiefang Avenue, Wuhan 430022, Hubei, China, Wuhan, Hubei, China (People’s Republic); Department of Geriatrics, Union Hospital, Tongji Medical College, Huazhong University of Science and Technology, 1277 Jiefang Avenue, Wuhan 430022, Hubei, China, Wuhan, Hubei, China (People’s Republic); Department of Geriatrics, Union Hospital, Tongji Medical College, Huazhong University of Science and Technology, 1277 Jiefang Avenue, Wuhan 430022, Hubei, China, Wuhan, Hubei, China (People’s Republic); Department of Geriatrics, Union Hospital, Tongji Medical College, Huazhong University of Science and Technology, 1277 Jiefang Avenue, Wuhan 430022, Hubei, China, Wuhan, Hubei, China (People’s Republic)

## Abstract

**Background:**

To investigate the association between physical, psychological, and social frailty, and to explore the mediating factors that affect frailty in elderly inpatients, while providing scientific evidence for frailty intervention.

**Methods:**

A cross-sectional study enrolled 134 hospitalized older patients with geriatric diseases. Based on the Fried frailty criteria, patients were categorized into physically frail and non-physically frail groups. Psychological frailty was assessed using the MMSE and GDS-15, while social frailty was evaluated with the WHOQOL-BREF and PSSS. Spearman’s correlation analyzed the relationship between the demographic variables and frailty. Mediation analyses elucidated relationships between frailty dimensions and their underlying mediators.

**Results:**

Age, self-care ability, risk of malnutrition, and comorbidity were significantly related with physical frailty. Meanwhile, psychological frailty (Cognitive impairment and depression) and social frailty (social support and quality of life) were positively correlated to physical frailty status. In addition, mediation analyses indicated that nutritional status mediated the relationship between psychological and physical frailty (MMSE: B = -0.0671, p < 0.0000; GDS-15: B = 0.0752, p < 0.0001), and between social and physical frailty (WHOQOL-BREF: B = -0.0229, p < 0.0000; PSSS: B = -0.0165, p < 0.0001).

**Conclusions:**

Physical frailty in elderly inpatients is associated with psychological and social frailty, with nutritional status mediating these relationships. Therefore, it is essential to conduct comprehensive assessments for the multidimensional aspects of frailty present in these patients, targeted interventions addressing malnutrition could disrupt the vicious cycle of multidimensional frailty.

